# Combined associations of a healthy lifestyle and body mass index with colorectal cancer recurrence and survival: a cohort study

**DOI:** 10.1007/s10552-023-01802-y

**Published:** 2023-10-02

**Authors:** Shabane Barot, Petri Rantanen, Caroline Nordenvall, Ulrik Lindforss, Åsa Hallqvist Everhov, Susanna C. Larsson, Annika Lindblom, Annelie Liljegren

**Affiliations:** 1 Department of Clinical Science and Education, Södersjukhuset, Karolinska Institutet, Stockholm, Sweden; 2 Department of Oncology, Södersjukhuset, 118 83 Stockholm, Sweden; 3 Department of Molecular Medicine and Surgery, Karolinska Institutet, Stockholm, Sweden; 4 Department of Pelvic Cancer, GI Oncology and Colorectal Surgery Unit, Karolinska University Hospital, Stockholm, Sweden; 5 Department of Surgery, Södersjukhuset, Stockholm, Sweden; 6 Unit of Medical Epidemiology, Department of Surgical Sciences, Uppsala University, Uppsala, Sweden; 7 Unit of Cardiovascular and Nutritional Epidemiology, Institute of Environmental Medicine, Karolinska Institutet, Stockholm, Sweden; 8 Department of Clinical Genetics, Karolinska University Hospital, Stockholm, Sweden; 9 Department of Oncology-Pathology, Karolinska Institutet, Stockholm, Sweden; 10 Department of Internal Medicine, Södersjukhuset, Stockholm, Sweden

**Keywords:** Colorectal neoplasms, Epidemiology, Life style, Survival analysis

## Abstract

**Purpose:**

Colorectal cancer (CRC) risk is associated with modifiable lifestyle factors including smoking, physical inactivity, Western diet, and excess body weight. The impact of lifestyle factors on survival is less known. A cohort study was conducted to investigate the combined effects of a healthy lifestyle and body mass index on prognosis following CRC diagnosis.

**Methods:**

Treatment and follow-up data were collected from the patient files of 1098 participants from the Colorectal cancer low-risk study cohort including stage I-III CRC patients. A healthy lifestyle and BMI (HL) score was computed using self-reported data on smoking status, physical activity, adherence to a Mediterranean diet pattern, and BMI, and divided into four categories ranging from least to most healthy. Survival analyses were performed to assess recurrence-free survival and overall survival across categories of exposure, using the Kaplan–Meier method and Cox proportional hazards models adjusted for age, sex, and educational level.

**Results:**

Among 1098 participants with stage I-III CRC, 233 (21.2%) had an HL score of 0–1 (least healthy), 354 (32.2%) HL score of 2, 357 (32.5%) HL score of 3 and 154 (14.0) HL score 4 (most healthy). Patients with the healthiest lifestyle (HL score 4) compared to the least healthy (HL score 0–1) had an improved recurrence-free survival (HL 4 vs HL 0–1, HRadj 0.51 (95% CI 0.31–0.83) and overall survival (HL 4 vs HL 0–1, HRadj 0.52 (95% CI 0.38–0.70).

**Conclusion:**

Adherence to a healthy lifestyle may increase the recurrence-free and overall survival of patients with stage I–III CRC.

**Supplementary Information:**

The online version contains supplementary material available at 10.1007/s10552-023-01802-y.

## Background

Colorectal cancer (CRC) is the third most common cancer globally, with incidence rates that positively correlate with Human Development Index levels (HDI), a measure of societal development based on the average health, education, and income of a population [1, 2]. Several CRC risk factors are associated with socioeconomic development including smoking, physical inactivity, unhealthy diets, and excess body weight [3]. Incidence rates are declining among older adults in high-income countries due to screening and early removal of precursor lesions but increasing among young adults (age < 50 years) [4]. The reason for this increase is unknown, but lifestyle exposures in childhood and adolescence are considered drivers [5]. Economically transitioning countries are seeing a rapid increase in CRC incidence, albeit from low levels, reflecting a global shift toward more Westernized lifestyles [6].

Less is known about the impact of lifestyle factors on CRC prognosis. Smoking [7, 8], physical inactivity [9, 10], and unhealthy diets [11, 12] have all been associated with increased mortality in CRC patients. The impact of overweight and obesity on CRC survival is, however, highly debated [13]. Some studies have reported improved survival among CRC patients with excess body weight compared to normal weight patients [14], the so-called obesity paradox [15]. Others report worse CRC-specific outcomes in the obese group [16, 17], and yet other studies find no associations between body mass index (BMI) and survival [18]. Reverse causality due to illness-induced weight loss may account for these differences, highlighting the importance of timing when assessing body weight [19].

Few studies have examined the combined effects of lifestyle factors on CRC recurrence and CRC-specific survival [20–23]. Previous studies have reported conflicting results, which may reflect methodological differences, including the timing of exposure assessment (pre/post-diagnosis), the use of different lifestyle scores, and differences in study populations. This study aimed to investigate the associations of the combined impact of pre-diagnostic modifiable healthy lifestyle factors, including avoidance of smoking, moderate to high levels of physical activity, high adherence to a healthy diet, and BMI within the healthy range, with CRC recurrence and overall survival in a cohort of Swedish CRC stage I-III patients.

## Methods

### Study design

The Colorectal cancer low-risk study cohort consists of more than 3300 participants diagnosed with all-stage CRC in 14 hospitals in Middle Sweden from 2003 to 2009, as described elsewhere [24]. Participants were consecutively recruited or identified using data provided by Regional Oncologic Centers. The latter received letters of invitation to participate in the study, and those interested were contacted over the telephone for informed consent and inclusion. A subset of participants included in 2004–2006 received a self-administered questionnaire on lifestyle habits (*n* = 1767), with a response rate of 93% (*n* = 1639).

We conducted a cohort study including participants from the Colorectal cancer low-risk cohort with stage I–III CRC who had completed the lifestyle questionnaire. A healthy lifestyle was the exposure of interest and recurrence-free survival (RFS) was the primary outcome of this study, using overall survival (OS) as a secondary outcome.

### Participants

Participants with a radically resected adenocarcinoma of the colon or rectum that had surgery in 2003–2006 were eligible for inclusion. Stage IV CRC patients were excluded due to dismal prognosis, as were participants with unavailable patient files or missing data on the American Joint Committee of Cancer (AJCC) TNM stage. Patients were staged according to version 5 of the AJCC TNM [25].

Two investigators (S.B and P.R) collected treatment and follow-up data for the participants during the years 2017–2020, including date of surgery, American Society of Anesthesiologists (ASA) classification, oncological treatment (neoadjuvant radiotherapy/adjuvant chemotherapy), time to CRC recurrence, time to last recurrence-free follow-up visit, and time to all-cause death.

### Exposure assessment

A semiquantitative questionnaire was used for the collection of information on smoking, physical activity, and anthropometric markers. Participants were asked to report their cigarette smoking status and history including the number of cigarettes per day and duration of smoking for current and ever-smokers. Data on physical activity including leisure time exercise was collected using a validated set of questions with 5 pre-defined duration categories ranging from less than 1 h to more than 5 h/week, a validated method for assessing physical activity [26]. Self-reported weight 5 years before diagnosis was used to calculate BMI by dividing the weight in kilograms by the square of height in meters. Weight 5 years prior to diagnosis was chosen to minimize the risk of reverse causation, as CRC can induce weight loss.

The lifestyle questionnaire included a food frequency section designed to assess a typically Swedish diet. Participants were asked to report serving size and average intake frequency of 96 commonly eaten foods and beverages 5 years before diagnosis. A similar validated questionnaire, where participants report eating habits over the last year, has been used in previous studies [27, 28].

#### Mediterranean diet score

The Mediterranean diet (MD) is one of the most scientifically evaluated dietary patterns in the field of nutritional epidemiology [29, 30]. Several studies have reported inverse associations between MD adherence and CRC risk and mortality [31–35]. A diet adhering to the MD pattern was considered healthy in this study.

We used the modified Mediterranean diet scale (mMED) defined by Tektonidis et al. and developed further by Larsson et. al to compute a diet variable [36, 37]. This is a modification of the Mediterranean diet scale originally constructed by Trichopoulou, to better suit the intake habits of the Swedish population. The mMED score was created by categorizing the intakes of the following 6 food groups into quintiles: vegetables and fruits, legumes and nuts, whole grains, fish, dairy products, and red and processed meats. Participants received a score from 1 to 5 for being in the first five groups’ lowest to highest quintiles of intake. The score was reversed for the last group, red and processed meats, assigning 5 points to the lowest quintile. The use of olive- or rapeseed oil was assigned 5 points; conversely, 1 point was assigned for non-use. The mMED has previously included intakes of alcoholic beverages. However, the health effects of moderate alcohol consumption are widely debated [38], prompting us to exclude alcohol consumption from the score. The total mMED score thus ranged from 7 (low adherence) to 35 (high adherence).

#### Healthy lifestyle and BMI score

A healthy lifestyle and BMI (HL) score was created by dichotomizing each of the four lifestyle variables into a pre-defined healthy and less healthy/unhealthy alternative [39]. Never smokers and former smokers with > 1 year of cessation time were considered non-smokers, as opposed to current and former smokers with ≤ 1 year of cessation. Current but not former smoking has been associated with poorer CRC-specific survival [7]. According to the WHO recommendations for adults, participants with ≥ 150 min/week of leisure time exercise were considered physically active, versus < 150 min/week [40]. A low-risk diet was defined as an mMED score > the cohort median, versus an mMED score ≤ cohort median. Participants with a BMI of 18.5–24.9 were considered to have healthy body weight, as opposed to those with underweight (BMI < 18.5) and pre-obesity or obesity (BMI ≥ 25.0 m^2^), according to the WHO classification [41]. One point was allocated for each healthy lifestyle factor, and 0 points for the less healthy or unhealthy alternative. The total score thus ranged from 4 (most adherent to a healthy lifestyle) to 0 (least adherent).

### Outcome assessment

CRC recurrence was defined as locoregional recurrence, distant metastasis, or the occurrence of a new colorectal tumor. Observation time started on the date of curative surgery and ended on recurrence or the date of the last follow-up visit to the surgical or oncological clinic. In the OS analysis, participants were observed from the date of curative surgery to the date of all-cause death or the last known date of contact.

### Statistical methods

We categorized participants into four groups based on HL points. Those with an HL score of 0 and 1 were combined into one group due to low numbers in the former category (*n* = 20). Those missing data on smoking (0.8%) were coded as non-smokers. Participants who had left the entire diet section of the FFQ blank were considered non-responders and excluded (0.9%). Median imputation was used to replace missing values for single food groups (6.5%), physical activity (2.8%), and BMI (2.6%).

The distribution of demographic variables across categories of exposure was tested using the chi-square test for categorical and the Kruskal–Wallis test for continuous variables. We used the Kaplan–Meier (K-M) method to assess median RFS and OS in each group and Cox proportional hazards model analysis to estimate univariate and multivariable-adjusted hazard ratios (HRs) and 95% confidence intervals (CIs) for CRC recurrence and all-cause death. The survival analysis was right censored. The least healthy group served as the reference category.

The pre-defined confounders age, sex, and educational level were included in the multivariable model (Figure S1). Tumor stage, oncological treatment, and tumor site were considered potential mediators of the effect of a healthy lifestyle on RFS (Figure S1). Diabetes and cardiovascular disease (CVD) were considered potential mediators of the effect of a healthy lifestyle on OS (Figure S2).

Using the Wald test, we tested for interactions between the HL score and tumor site, oncological treatment, and tumor stage. Since most participants with rectal cancer had received neoadjuvant radiotherapy (RT) before surgery, that is before the start of observation time, we analyzed rectal cancer patients separately using an RT variable as a covariate in the multivariate regression model. A complete cases-only analysis was conducted excluding those missing data on any of the HL score variables.

All analyses were done using SPSS version 28.

## Results

**Fig. 1:**
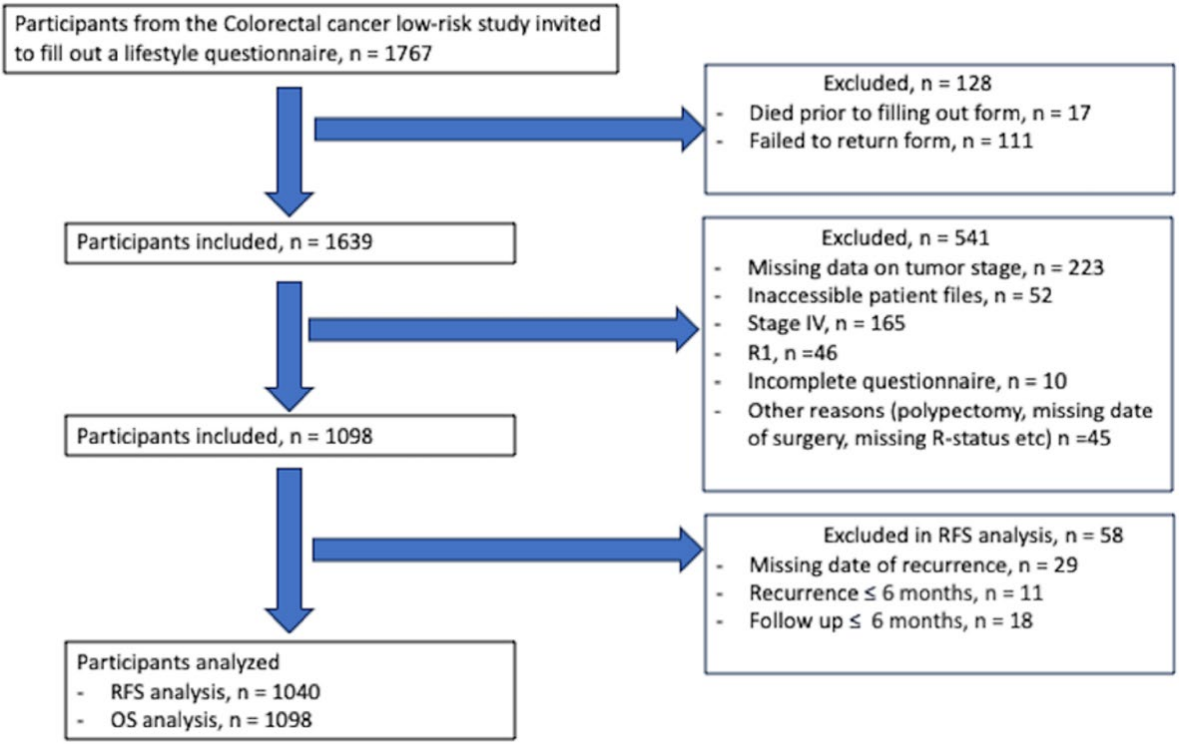
Flow diagram of participants illustrating the study design according to Strengthening the Reporting of Observational Studies in Epidemiology (STROBE) recommendations. *N*  number, *CRC*  colorectal cancer, tumor stage = stage according to AJCC TNM 5th edition, *R1* microscopically positive resection margins, *RFS* recurrence-free survival, *OS* overall survival, *m* months

Participants missing an exact date of recurrence (*n* = 29), with recurrences occurring ≤ 6 months of diagnosis (*n* = 18) or follow-up time ≤ 6 months (*n* = 11) were excluded from the RFS analysis (*n* =58 ). A total of 1040 participants were included in the RFS analysis and all 1098 participants were included in the OS analysis (Fig. 1).

Demographic characteristics of participants are shown for the total population and by HL score category in Table 1.

**Table 1:** Characteristics of 1098 patients with stage I–III colorectal cancer in the Colorectal cancer low-risk cohort, for the population as a whole and by Healthy lifestyle and Body Mass Index score group

	All participants	HL 0–1 p*	HL 2 p	HL 3 p	HL 4 p
Participants, *n* (%)	1098	233 (21.2)	354 (32.2)	357 (32.5)	154 (14.0)
Age at colorectal cancer diagnosis in years, median (IQR**)	69 (62–77)	67 (60–75)	69 (62–77)	70 (63–77)	71 (65–79)
*Sex*					
Men, *n* (%)	573 (52.2)	127 (54.5)	189 (53.4)	199 (55.7)	58 (37.7)
Women, *n* (%)	525 (47.8)	106 (45.5)	165 (46.6)	158 (44.3)	96 (62.3)
ASA class***					
1, *n* (%)	245 (22.3)	50 (21.5)	73 (20.6)	81 (22.7)	41 (26.6)
2, *n* (%)	648 (59.0)	133 (57.1)	215 (60.7)	211 (59.1)	89 (57.8)
3, *n* (%)	193 (17.6)	48 (20.6)	62 (17.5)	59 (16.5)	24 (15.6)
4, *n* (%)	12 (1.1)	2 (0.9)	4 (1.1)	6 (1.7)	0 (0.0)
*Year of diagnosis*					
2003, *n* (%)	59 (5.7)	16 (6.9)	16 (4.5)	22 (6.2)	5 (3.2)
2004, *n* (%)	491 (44.7)	112 (48.1)	163 (46.0)	150 (42.0)	66 (42.9)
2005, *n* (%)	411 (37.4)	79 (33.9)	131 (37.0)	139 (38.9)	62 (40.3)
2006, *n* (%)	137 (12.5)	26 (11.2)	44 (12.4)	46 (12.8)	21 (13.6)
*Cancer site*					
Colon, *n* (%)	696 (63.4)	147 (63.1)	220 (62.1)	222 (62.2)	107 (69.5)
Rectum, *n* (%)	402 (36.6)	86 (36.9)	134 (37.9)	135 (37.8)	47 (30.5)
*TNM Stage according to AJCC*^a^					
I, *n* (%)	237 (21.6)	48 (21.5)	70 (19.8)	89 (24.9)	30 (19.5)
II, *n* (%)	462 (42.1)	100 (42.9)	157 (44.4)	135 (37.8)	70 (45.5)
III, *n* (%)	399 (36.3)	85 (36.5)	127 (35.9)	133 (37.3)	54 (35.1)
*Radiotherapy (only rectal cancer, total n = 403)*					
Rectal cancer patients treated with neoadjuvant RT, *n* (%)	296 (73.4)	61 (70.9)	109 (81.3)	97 (71.9)	29 (61.7)
*Postoperative Chemotherapy*					
Initiated, *n* (%)	236 (21.5)	53 (22.7)	78 (22.0)	75 (21.0)	30 (19.5)
Recieving 8 or more courses, *n* (%)	160 (14.6)	34 (14.6)	47 (13.3)	58 (16.2)	21 (13.6)
*Cardiovascular morbidity*					
Severe^b^, *n* (%)	179 (16.3)	37 (15.9)	52 (14.7)	68 (19.0)	22 (14.3)
Hypertension, *n* (%)	315 (28.7)	75 (32.2)	107 (30.2)	89 (24.9)	44 (28.6)
Diabetes, *n* (%)	161 (14.7)	49 (21.0)	49 (13.8)	48 (13.4)	15 (9.7)
*Acetylsalicylic acid*					
Regular consumers, *n* (%)^c^	174 (15.8)	38 (16.3)	54 (15.3)	56 (15.7)	26 (16.9)
*Alchohol consumption*					
High risk consumers, *n* (%) ^d^	173 (15.8)	38 (16.3)	57 (16.1)	56 (15.7)	22 (14.3)
*Educational level* ^e^					
9 years, *n* (%)	704 (64.1)	163 (70.0)	242 (68.4)	213 (59.7)	86 (55.8)
12 years, *n* (%)	141 (12.8)	30 (12.9)	45 (12.7)	52 (14.6)	14 (9.1)
> 12 years, *n* (%)	253 (23.0)	40 (17.2)	67 (18.9)	92 (25.8)	54 (35.1)
*Follow-up time in years * DFS, median, years (95% CI)	4.3 (4.1–4.5)	3.8 (3.3–4.2)	4.8 (4.3–5.2)	4.3 (4.0–4.6)	4.1 (3.7–4.6)
OS, median, years (95% CI)	6.3 (6.0–6.6)	6.0 (5.4–6.5)	6.7 (6.2–7.2)	6.0 (5.5–6.4)	6.8 (6.0–7.5)
Minimum follow-up time – maximum follow-up time, years	0.6–18.1	0.8–14.8	0.7–18.2	0.6–18.2	0.8–16.3
Relapse, *n* (%)	221 (20.3)	65 (27.9)	63 (17.8)	69 (19.3)	24 (15.6)

*Healthy lifestyle and Body Mass Index score groups, 0–1 points, 2 points, 3 points, 4 points

**IQR  Interquartile range

***ASA  American Society of Anesthesiologists Physical Status Classification system

^a^*AJCC*  American Joint Committee on Cancer, *TNM*  Tumor, Node, Metastasis classification

^b^Severe cardiovascular morbidity = stroke, acute myocardial infarction, angina pectoris

^c^Regular consumers are defined as those using ≥ 7 tablets of acetylsalicylic acid /week

^d^≥ 9 units/week for women, ≥ 12 for men

^e^Highest completed level of education, 9 years = grade school, 12 years = high school, > 12 years = university

The group with the healthiest lifestyle (HL 4) consisted of 157 participants (14%). These were more likely to be women, of higher age, with a higher educational level, and less often diabetics, as compared to the 233 participants (21%) in the least healthy group (HL 0–1) who were predominantly male and tended to be younger at CRC onset. There were no differences in cancer stage, tumor site, oncological treatment, or other clinical factors. The composition of the HL score is further outlined in Table 2.

**Table 2:** Healthy Lifestyle and Body Mass Index Score composition, showing the distribution of score components for the study population as a whole and by score groups

	Points	All participants	0–1 p	2 p	3 p	4 p
*Smoking*						
Current + former < 1 y**, *n* (%)	0	139 (12.7)	71 (30.5)	47 (13.3)	21 (5.9)	0 (0.0)
Never + former ≥ 1 y, *n* (%)	1	959 (85.3)	162 (69.5)	307 (86.7)	336 (94.1)	154 (100)
*Physical activity, strenuous*						
< 150 min/week, *n* (%)	0	434 (39.5)	218 (93.6)	162 (45.8)	54 (15.1)	0 (0.0)
≥ 150 min/week *n* (%)	1	664 (60.5)	15 (6.4)	192 (54.2)	303 (84.9)	154 (100)
BMI** (kg/m^*2*^*)*						
< 18.5, *n* (%)	0	13 (1.2)	4 (1.7)	5 (1.4)	4 (1.1)	0 (0.0)
18.5–24.9, *n* (%)	1	484 (44.1)	28 (12.0)	105 (29.7)	197 (55.2)	154 (100)
25.0–29.9, *n* (%)	0	470 (42.8)	141 (60.5)	197 (55.6)	132 (37.0)	0 (0.0)
≥ 30, *n* (%)	0	131 (11.9)	60 (25.8)	47 (13.3)	24 (6.7)	0 (0.0)
mMED***-adherence						
mMED score ≤ 20, *n* (%)	0	599 (54.6)	227 (97.4)	250 (70.6)	122 (34.2)	0 (0.0)
mMED score > 20, *n* (%)	1	499 (45.4)	6 (2.6)	104 (29.4)	235 (65.8)	154 (100)

*p  points

**y  year

***BMI  body mass index

***mMED  modified Mediterranean diet score

**Fig. 2:**
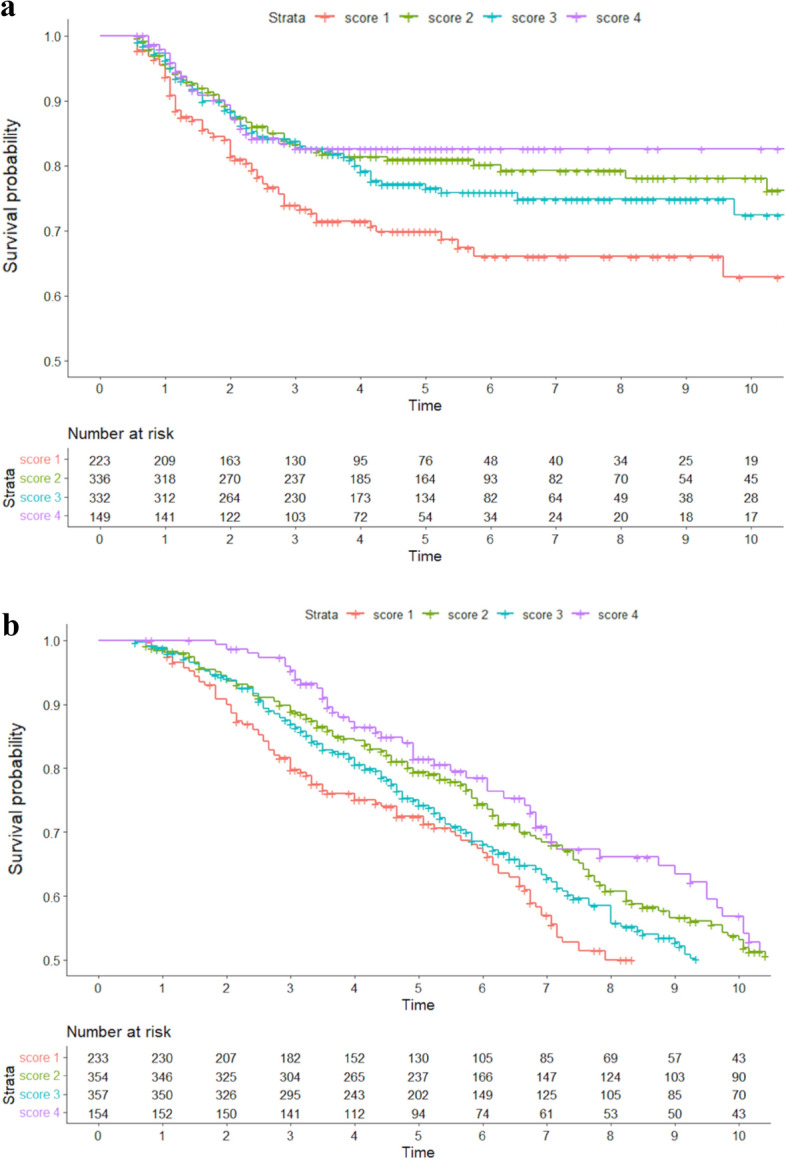
**a **and** b** Kaplan–Meier estimates for **a** recurrence-free survival and **b** overall survival by Healthy lifestyle and Body Mass Index score groups among patients with stage I-III colorectal cancer. Time since curative surgery is expressed in years

We observed 221 events of cancer recurrence among 1040 participants during a median follow-up time of 4.3 years (Fig. 2).

A healthy lifestyle was associated with improved RFS. The crude and adjusted HRs of recurrence and death were significantly lower than the reference for all score categories above the reference. Compared to participants with an HL 0–1 (least healthy), the HL 4 (most healthy) category had a crude HR for recurrence of 0.51 (95% CI 0.32–0.81) and an adjusted HR for recurrence of 0.51 (95% CI 0.31–0.83), with sex, age, and educational level included in the multivariate model. The adjusted HRs for recurrence of participants with an HL 2 and HL 3 were 0.57 (95% CI 0.40–0.81) and 0.66 (0.47–0.92), respectively (Table 3). There were 542 deaths among 1098 participants during a median follow-up time of 6.3 years (Fig. 2). The crude HR for all-cause death for participants with HL 4 vs HL 1 was 0.65 (95% CI 0.48–0.87) and the adjusted HR for all-cause death was 0.52 (95% CI 0.38–0.70) (Table 3). The adjusted HRs for death of HL 2 and 3 vs HL 1 were 0.66 (0.50–0.79) and 0.72 (0.57–0.90). We found no significant interactions between the covariates included in the multivariate model when using the Wald test.

**Table 3:** Univariate and Multivariate survival analysis using Cox proportional hazards regression models for colorectal cancer recurrence and overall death by Healthy lifestyle and BMI score

HL score**	Recurrences, *n*^a^	Deaths, *n*	Univariate				Multivariate			
			CRC* recurrence		All-cause death		CRC* recurrence		All-cause death	
			HR	95% CI^c^	HR^b^	95% CI	HR	95% CI	HR	95% CI
0–1 p	65	132	1 (ref***)		1 (ref)		1 (ref)		1 (ref)	
2 p	60	164	0.57	0.40–0.81	0.70	0.55–0.67	0.57	0.40–0.81	0.63	0.50–0.79
3 p	72	177	0.66	0.47–0.92	0.84	0.67–1.06	0.66	0.47–0.93	0.72	0.57–0.90
4 p	24	69	0.51	0.32–0.81	0.65	0.48–0.87	0.51	0.31–0.83	0.51	0.38–0.70

Multivariate model adjusted for age, sex, and educational level

*Colorectal cancer

**Healthy lifestyle and BMI score

***reference

^a^Number

^b^Hazard ratio

^c^Confidence interval

In the K-M curves for recurrence (Fig. 2), the curve of the least healthy group was found to have a disproportional course in relation to the others with events occurring sooner in this group, indicating changes in HR over time. The proportional hazards assumption was not valid (Log-Rank test *p*-value = 0.001). The HRs for recurrence are thus to be interpreted as average estimates for the whole time of observation.

### Sensitivity analysis

The violation of the proportional hazards assumption prompted us to conduct a Cox proportional hazards analysis with time-dependent covariates, in order to estimate the HRs for time periods < 24 months, ≥ 24 months – < 36 months, ≥ 36 months – < 48 months, and ≥ 48 months – < 60 months. The strongest effect of the HL score on RFS was seen in the interval of ≥ 24 months – < 36 months (Table S1).

We found no significant interaction effects between the HL score and cancer stage (*p*-value: 0.39), tumor location (*p*-value 0.68), and oncological treatment (*p*-value:0.66) when using the Wald test in the RFS analysis, A complete cases-only analysis was performed, excluding all cases with missing values in score components. This only slightly affected estimated HRs and 95% CIs (Table S2).

We analyzed the rectal cancer group separately, including a covariate for radiotherapy in the Cox model. Participants had received a total dose of either 25 Gy (80% of those treated with neoadjuvant RT) or 50,4 Gy. We coded a categorical variable with three levels (0 = no RT, 1 = 25 Gy, 2 = 50.4 Gy), and tested it in a Cox model for rectal cases only. The p-value of the RT-covariate was non-significant.

When including the individual score components as covariates in a multivariate Cox regression analysis, only smoking, and exercise were significantly associated with a reduced HR of CRC recurrence and death (Table S3). Sex, age, and level of education were all significantly associated with OS, but not RFS.

## Discussion

In this cohort study, patients with CRC stage I-III and a healthy lifestyle (HL 4) had a 49% lower HR of cancer recurrence and a 48% lower HR of all-cause death compared with the least healthy (HL 1). As we found the proportional hazards assumption to be violated in our survival analyses, indicating changes in hazard rate over time, we investigated the time-varying effects of the score on RFS. The results indicate a stronger effect in the interval of 24–36 months. However, only a small number of recurrences occurred after this period and the results for survival > 36 months should thus be interpreted with caution.

This is one of the first studies to report a statistically significant decrease in the risk of CRC recurrence in patients adhering to a healthy lifestyle pre-diagnosis. A small number of previous studies have reported inverse associations between a healthy lifestyle and overall mortality, but results for RFS or CRC-specific mortality have often been weaker.

Among 5727 all-stage CRC cases, Pelser et al. reported a statistically significant reduction in the risk of all-cause death for those with a pre-diagnostic healthy lifestyle (including a BMI within the normal range, smoking avoidance, physical activity, a healthy diet, and a low intake of alcohol) [20]. Reduced risk of CRC-specific death was seen only among rectal cancer cases, whereas our results indicate a protective effect of a healthy lifestyle irrespective of the anatomical subsite.

In a cohort of CRC patients from the Nurses’ Health Study (NHS) and the Health Professionals Follow-up Study (HPFS), pre-and post-diagnostic adherence to the World Cancer Research Fund/American Institute for Cancer Research (WCRF/AICR) score was significantly associated with a lower risk of all-cause, but not CRC-specific, death [21]. This score includes physical activity, diet, and body weight, but not smoking. Non-smoking was our study’s strongest individual risk-reducing factor, which may account for the differences. Among 3292 cases of all-stage CRC within the European Prospective Investigation into Cancer and Nutrition (EPIC) study, pre-diagnostic concordance with the WCRF/AICR recommendations was however associated with reduced CRC-related and overall mortality [22].

Among 992 colon cancer stage III cases, finally, a healthy lifestyle post-diagnosis (including normal body weight maintenance, physical activity, and a healthy diet) according to the guidelines issued by the American Cancer Society (ACS) was associated with a significant improvement in OS and a significant trend toward improved RFS over a 7-year median follow-up time [23]. Stratifying for tumor stage did not indicate stage-specific associations between lifestyle and survival in our study.

A recently published large study on the associations between healthy lifestyles and cancer morbidity and mortality in diabetics, including 1904 participants with CRC, found a 45% lower risk of cancer mortality among those with the healthiest lifestyle, compared to the least healthy [42]. Lifestyle and dietary factors have also been included in recurrence and survival prediction models for colon cancer stage III, resulting in significantly improved predictions [43].

The suggested biological mechanisms conferring a protective effect of a healthy lifestyle on CRC risk include decreases in inflammation and oxidative stress, modulation of gut microbiota, decreased bowel transit time, and increases in insulin sensitivity [44–46]. The same mechanisms may be involved in reducing the risk of recurrence. Traditional models of tumorigenesis have considered systemic tumor spread to be a late event in the process of primary tumor progression. This is being challenged by studies showing that dissemination can occur also in the early stages of this process [47, 48] even in preneoplastic lesions [49]. Environmental exposures during the process of tumorigenesis could thus influence the risk of dissemination.

We’ve used a diet score modified to suit the intakes of a Swedish population, which may impair the generalizability of our results. However, the other lifestyle variables were assessed using internationally established criteria and the results may thus apply to other high-income or even transitioning populations. Our results indicate that pre-diagnosis lifestyle has an impact not only on CRC risk but also on disease-specific survival, which underlines the importance of primary preventive measures. Further studies are warranted to confirm our results.

### Strengths and limitations

Our study has several strengths including a long follow-up time with many observed events, detailed clinical data, and a design that may have reduced the risk of reverse causation in lifestyle assessment. The study is based on high-quality questionnaires, and the method has been evaluated previously. Further, the proportion of questionnaire responders was high (93%) decreasing the risk of selection bias and missing data was scarce, increasing the internal validity. There are also weaknesses to consider, including the risk of misclassification bias in self-reported data. Unmeasured lifestyle changes post-diagnosis may be reflected in our results. Studies on lifestyle changes in CRC survivors report conflicting results, with some finding shifts towards more healthy dietary habits [50], and smoking cessation [51], while others report little or no change in lifestyle [52].

Our exposure variable, the HL score, is based on four dichotomized lifestyle factors, each given equal weight within the score. Our results however indicate that non-smoking and physical activity have a stronger association with an improved recurrence-free and overall survival than a healthy diet and BMI within the normal range. Future studies could thus consider using a weighted score. We chose to dichotomize the BMI variable, placing the underweight participants in the same “unhealthy” category as the overweight and obese. The impact of underweight, overweight, and obesity on CRC recurrence and survival may however differ, which should be considered in future studies. Using additional anthropometric markers may further improve body weight assessment [6]. Confounding due to additional unmeasured factors cannot be ruled out.

## Conclusions

Our study indicates that adherence to a healthy lifestyle may increase the RFS and OS of patients with stage I-III CRC. Avoidance of smoking and being physically active were independent risk-reducing factors for these outcomes.

### Supplementary Information

Below is the link to the electronic supplementary material.

Supplementary file1 (DOCX 4549 KB)

## Data Availability

The datasets analysed during the current study include sensitive and detailed data on the health and habits of study participants. They are not publicly available due to the risk of compromising their anonymity, but available from the corresponding author on reasonable request.
