# Supplementary material for: Combined associations of a healthy lifestyle and body mass index with colorectal cancer recurrence and survival: a cohort study
Source: Cancer Causes Control. 2023 Oct 2;35(2):367–76. doi: 10.1007/s10552-023-01802-y (PMC10787671; doi:10.1007/s10552-023-01802-y)

Table S1: Multivariate recurrence-free survival analysis using Cox proportional hazards regression models with time-dependent covariates. Adjusted for sex, age, and educational level.

| **Recurrence-free survival** | | | | | | | | |  |
| --- | --- | --- | --- | --- | --- | --- | --- | --- | --- |
| **< 24 months** | | **≥ 24 months – <36 months** | | **≥ 36 months – < 48 months** | | | **≥ 48 months – <60 months** | |  |
| **HL* score** | HR** | 95% CI*** | HR | 95% CI | | HR | 95% CI | HR | 95% CI |
| **1** | 1 (ref¨) |  | 1 (ref) |  | | 1 (ref) |  | 1 (ref) |  |
| **2** | 0.63 | 0.39-1.01 | 0.52 | 0.27-1.00 | | 0.80 | 0.22-2.80 | 0.25 | 0.02-2.74 |
| **3** | 0.69 | 0.43-1.10 | 0.45 | 0.23-0.91 | | 1.26 | 0.39-4.09 | 1.70 | 0.34-8.41 |
| **4** | 0.64 | 0.34-1.18 | 0.57 | 0.25-1.32 | | 0.32 | 0.04-3.00 | - | - |

* Healthy lifestyle a BMI score

** Hazard ratio

*** confidence interval

¨reference

Table S2: Multivariate recurrence-free survival analysis using Cox proportional hazard models, including 980 complete cases only Adjusted for age, sex, and educational level.

| **Recurrence-free survival**  n= 980 | | |
| --- | --- | --- |
| **HL* score** | HR** | 95% CI*** |
| 1 | *referent* |  |
| 2 | 0.49 | 0.33-0.72 |
| 3 | 0.60 | 0.41-0.88 |
| 4 | 0.50 | 0.31-0.81 |

* Healthy lifestyle and BMI score

** Hazard ratio

*** Confidence interval

Table S3: Multivariate recurrence-free survival analysis using Cox proportional hazard models including individual score components. Adjusted for age, sex, and educational level.

| **Recurrence-free survival** | | |
| --- | --- | --- |
|  | HR* | 95% CI** |
| Smoking | *referent* |  |
| Non-smoking | 0.62 | 0.43-0.90 |
|  |  |  |
| Physical exercise <150 min | *referent* |  |
| Physical exercise  ≥ 150 min | 0.71 | 0.54-0.94 |
|  |  |  |
| mMED^ non-adherence | *referent* |  |
| mMED adherence | 0.95 | 0.72-1.25 |
|  |  |  |
| BMI¨ < 18.5 or ≥ 25 | *referent* |  |
| BMI 18.5-24.9 | 0.97 | 0.73-1.29 |

* Hazard ratio

** Confidence interval

^ modified Mediterranean diet

¨Body mass index

**Figure S1:** Direct acyclic graph depicting potential mediators and cofounders in the relationship of exposure, a healthy lifestyle and outcome, and recurrence-free survival. **(Supplementary)**


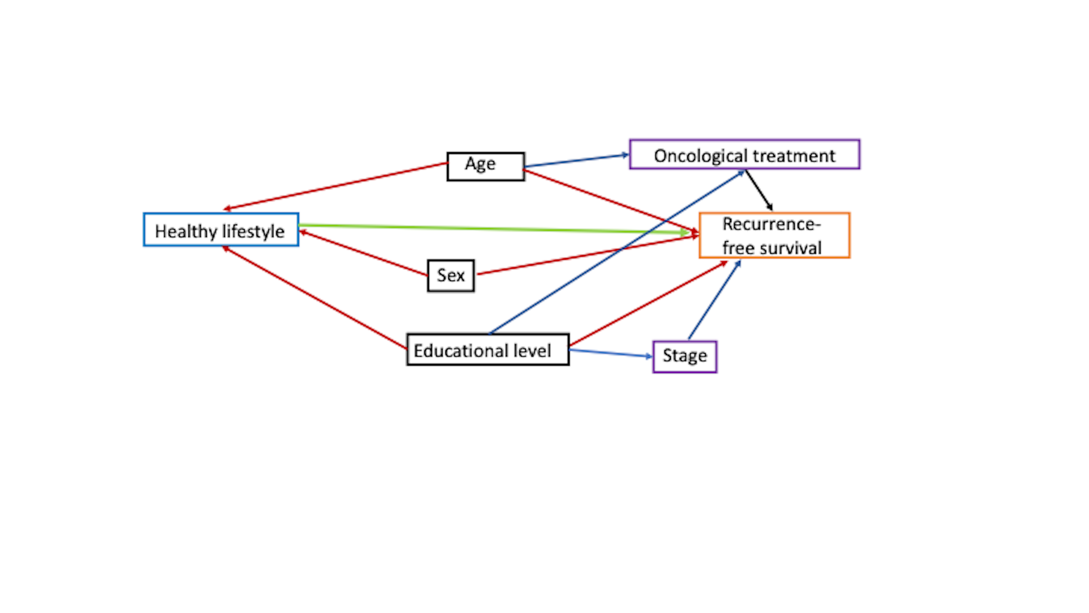


**Figure S2:** Direct acyclic graph depicting potential mediators and cofounders in the relationship of exposure, a healthy lifestyle and outcome, and overall survival. **(Supplementary) .**


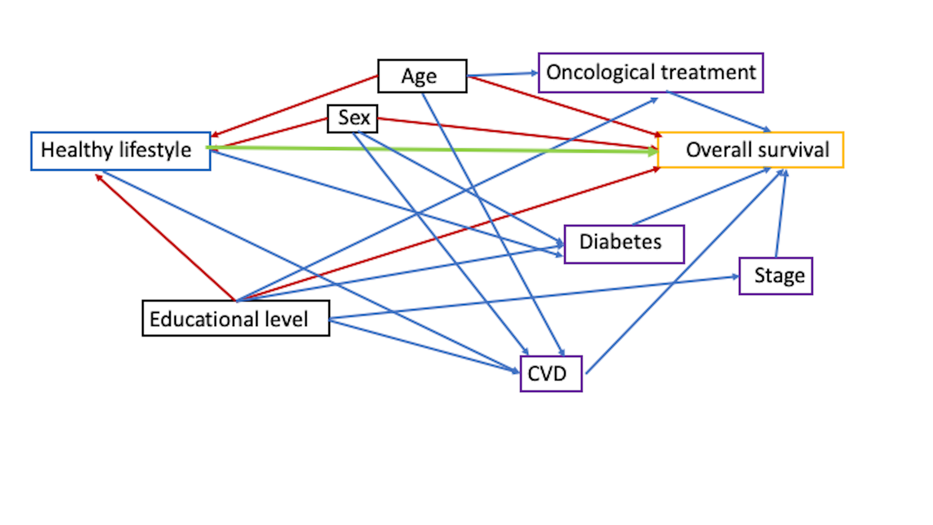

Supplement: Supplementary file 1 — Supplementary file1 (DOCX 4549 KB) [file 10552_2023_1802_MOESM1_ESM.docx]
